# Exploring the use of mouth guards in Muay Thai: a questionnaire survey

**DOI:** 10.1038/s41405-020-00048-z

**Published:** 2020-10-15

**Authors:** Kimberley Pickering, Susan M. Bissett, Richard Holliday, Christopher Vernazza, Philip M. Preshaw

**Affiliations:** 1 School of Dental Sciences, Newcastle University, Newcastle upon Tyne, UK; 2 National University Centre for Oral Health, National University of Singapore, Singapore, Singapore

**Keywords:** Oral diseases, Dental trauma

## Abstract

**Objective:**

To identify Muay Thai participants’ attitudes towards use of mouth guards and their experiences of dental trauma.

**Materials and methods:**

An online cross-sectional survey was used to record Muay Thai participants’ experiences and opinions regarding use of mouth guards. Participants were recruited from a Muay Thai gym in the north east of England.

**Results:**

92 respondents took part in the survey. 3% reported having never worn a mouth guard, whereas 61% reported routinely wearing mouth guards during a fight. Significantly more (73%) younger participants (18–29 years) reported wearing mouth guards during fights compared to those aged 30 years and older (50%) (*p* < 0.05). Mouth-formed (‘boil and bite’) were the most frequently used type of mouth guard (60% of users), followed by custom-made mouth guards provided by a dentist (32%). Factors such as protection, breathing, good fit and comfort were all considered important in the choice of mouth guard. 14% of respondents had experienced dental injuries, with chipped/broken teeth being the most common.

**Conclusion:**

Given the risk for dental trauma in Muay Thai, it is important that participants are advised regarding mouth guard use, particularly those that do not routinely wear them.

## Introduction

Muay Thai is an aggressive and high risk contact sport, a physical and mental discipline which involves fighting with fists, feet, knees and elbows. It is rapidly increasing in popularity, with an estimated one million participants worldwide, and in the UK alone there are more than 120 nationally registered Muay Thai gyms with an estimated 10,000 participants.^1^ It has been shown that head and facial injuries were the second most common types of injury among amateur and professional Muay Thai participants, and the nature or severity of injury may depend on the ability level of the individual.^2^ A mouth guard is a recommended piece of equipment for protecting the mouth when participating in a contact sport such as Muay Thai.^3^ Mouth guards help protect from an impact that could potentially cause dental trauma, oral soft tissue injuries, bone fractures or dislocation, or lacerations to the lips.^4^

There are three main types of mouth guards in routine use. Prefabricated (or stock) mouth guards are the simplest variety, and can be used ‘off the shelf’, but often suffer from poor fit. Mouth-formed (or ‘boil and bite’) mouth guards are heated (e.g. in hot water) to soften the plastic, and then formed (moulded) inside the user’s mouth. The third type are custom-made mouth guards, which require an impression to be taken by a dental practitioner and are fabricated using vacuum-formed ethylene vinyl acetate or similar.^5^ Whereas mouth guards are recommended in many Muay Thai gyms, their use is not always mandatory.

Previous studies into Muay Thai injuries have paid limited attention to dental trauma.^2,6–11^ However, in a study of 260 Muay Thai participants in Thailand, it was noted that 23.5% had experienced dental and jaw injuries.^12^ Furthermore, in a study of 120 males participating in contact sports (including Muay Thai) in Iran, it was identified that 95 had suffered at least one traumatic injury to the face requiring medical treatment, with 53 of the participants experiencing dental injuries (including displacement, luxation, fracture and avulsion).^13^ The authors of this study reported that different combat sports have differing injury profiles, with maxillofacial injury rates being twice as high among professional (86%) as opposed to amateur (42%) athletes.

Given the reported high prevalence rates of dental trauma among combat sports participants, the aim of this study was to identify Muay Thai participants’ attitudes towards use of mouth guards and their experiences of dental trauma.

## Materials and methods

This study was a cross-sectional survey using an anonymously completed questionnaire. An online tool was used to disseminate the questionnaire and collect responses. The questionnaire was designed according to previous recommendations and included a mix of quantitative and open-ended response items.^14^ Following initial development of the questionnaire, it was reviewed by two public participant groups (the Newcastle University Oral and Dental Patient Carer and Public Involvement (PCPI) group and a specifically established Muay Thai participant group) and piloted before use. The questionnaire grouped items into three sets: (i) basic personal and demographic information; (ii) individual experiences, including involvement in Muay Thai, use of mouth guards and dental health issues; and (iii) opinions regarding mouth guards and their use. Responses were either multiple choice categorical data, Likert scale data, or open-ended free text responses. Items about perceptions and opinions addressed different aspects of risk taking, responsibility, influences and changes of behaviour following injury.

The questionnaire was distributed by posting an invitation on the Facebook site of a Muay Thai gym in Newcastle upon Tyne, UK, and then relying upon snowball recruitment. This gym was selected because its members included a broad spread of beginners, advanced participants, coaches and professionals, with the aim to receive responses from a wide range of individuals at different experience levels. Respondents were also encouraged to forward the invitation to Muay Thai participants from other gyms. The invitation included a link allowing the questionnaire to be activated, filled in and returned electronically. Response data were collected and stored by the questionnaire tool and subsequently exported for analysis. Two follow-up reminders were posted over the first two weeks of data collection, and the survey was closed after four weeks.

Data were exported into SPSS (Statistical Package for the Social Sciences version 23.0; SPSS Inc. Chicago, USA) for analysis. Frequency distributions were computed for individual category and Likert scale responses to identify the most common responses and the variation in responses. Cross tabulations were used to analyse possible associations between selected responses and significance testing was performed using chi-squared tests. Given the lack of previous information on this topic, a power calculation could not be performed. However, we aimed to achieve a response of approximately 100 participants and considered that this would provide meaningful data in this exploratory study.

Ethical approval was obtained from the Newcastle University Research Ethics Committee (reference 7899/2018) prior to study commencement.

## Results

Ninety-two completed questionnaires were received. Given that answers to some of the items were optional depending on earlier responses, the *N* values for some items do not sum to 92. The study population comprised 66 males (71.7%) and 26 females (28.3%). With regards to age, 24 (26.1%) were 18–24 years, 20 (21.7%) were 25–29 years, 13 (14.1%) were 30–34 years, 16 (17.4%) were 35–39 years and 19 (20.7%) were 40 years or older. Table 1 presents data regarding participants’ involvement in Muay Thai. There was a high representation of advanced fighters and above (78.3% of the study population), with many participants having an extensive training history (e.g. 28.2% reported training for 10 or more hours per week, and 37.0% had been active in Muay Thai for 10 years or more). Over one-third of respondents (41.3%) reporting having 10 or more inter-club or competition fights, and almost half of respondents (44.6%) competed nationally or internationally.

**Table 1:** Characteristics of participants’ involvement in Muay Thai.

Experience level	Beginner	Advanced	Professional	Coach
*N* (%)	20 (21.7%)	53 (57.6%)	9 (9.8%)	10 (10.9%)
Hours’ participation per week	1–4	5–9	10–20	>20
*N* (%)	35 (38.0%)	30 (32.6%)	21 (22.8%)	5 (5.4%)
Years active	<1	1–4	5–9	≥10
*N* (%)	7 (7.6%)	29 (31.5%)	21 (22.8%)	34 (37.0%)
Competition level	Sparring	Inter-club	National	International
*N* (%)	14 (15.2%)	24 (26.1%)	34 (37.0%)	7 (7.6%)
N of competition fights	None	1–4	5–9	≥10
*N* (%)	12 (13.0%)	23 (25.0%)	19 (20.7%)	38 (41.3%)

With regards to mouth guard use, just three participants (3.3%) reported having never worn a mouth guard. Eighty-one participants (88.0%) reported wearing a mouth guard during sparring (training sessions against an opponent with physical contact, though not classified as a fight), whereas fewer respondents (*n* = 56, 60.9%) reported wearing mouth guards during a fight (which could be a fight within a routine gym training session, or during a competition). Four participants (4.3%) also wore a mouth guard during warm-up sessions (sessions with no physical contact against an opponent). Mouth-formed (‘boil and bite’) mouth guards were the most frequently used (59.8% of users), followed by custom-made mouth guards provided by a dentist (31.5%). Just 3.3% of participants were using prefabricated (stock) mouth guards.

**Fig. 1:**
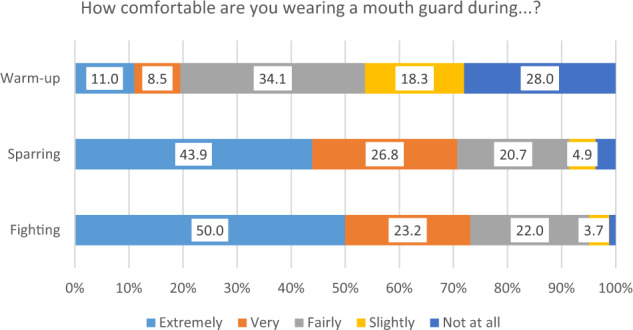
Stacked bar chart to show participant responses to the question ‘How comfortable are you wearing a mouth guard during warm-up, sparring and fighting?’.

**Fig. 2:**
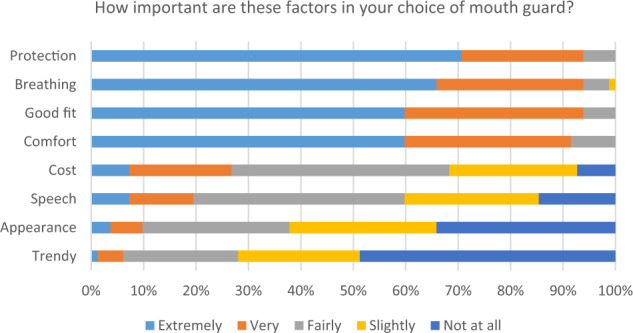
Stacked bar chart to show participant responses regarding factors considered important in their choice of mouth guard.

A large proportion of participants reported being either extremely or very comfortable wearing a mouth guard during sparring (70.7%) or fighting (73.2%), but less so during warm-up (19.5%) (Fig. 1). The great majority of respondents indicated that protection against injury, breathing, good fit and comfort were either extremely or very important factors in their choice of mouth guard (>90% for all) whereas factors such as cost, speech, appearance, or the trendiness of mouth guards were less important (Fig. 2). With respect to the cost of their mouth guards, all of those using prefabricated stock mouth guards reported paying less than £20. Of those using mouth-formed (‘boil and bite’) mouth guards, the majority (83.6%) reported paying less than £20, 12.7% reported paying between £20 and £50, and 3.6% had been given theirs for free (or were sponsored). For those using custom-made mouth guards provided by a dentist, 17.2% were free/sponsored, 3.4% cost less than £20, 37.9% cost £20–£50, and 41.4% cost more than £50.

When comparing mouth guard use between younger (18–29 years, *n* = 44), and older (30 years or more, *n* = 48) participants, bivariate analyses revealed no significant differences in usage when warming up or sparring (*p* > 0.05). However, a statistically significant difference in mouth guard use during fights was observed, with 32 of the 18–29 year-olds (72.7%) reporting usage, as compared to 24 (50.0%) of those aged 30 years or more (chi-squared test, *p* = 0.026).

Just 36% of respondents were aware of regulations or advice on wearing mouth guards in Muay Thai. 80% of respondents were currently registered with a dentist and 8% were not registered (with the remainder not recording a response to this question). 41 of the respondents had told their dentist that they participated in Muay Thai. Of these, 12% reported having taken their mouth guard to the dentist so that it could be checked, and 73% had been recommended a custom-made mouth guard by their dentist. Regarding the full study population, 57% would definitely recommend using a custom made mouth-guard from a dentist. Respondents agreed overwhelmingly (84%) that mouth guards should be mandatory for children in Muay Thai, and the majority of respondents (62%) agreed that participants should take the primary responsibility for ensuring that a mouth guard is used, followed by coaches (11%) and governing bodies (10%).

13 respondents (14%) reported having suffered dental injuries in Muay Thai. Of these, the most frequently reported injuries were chipped/broken teeth (*n* = 10), broken crown (*n* = 1), tooth avulsion (*n* = 1, reported as a primary tooth that was loose anyway), and locked jaw (*n* = 1). When asked if the experience of dental injuries had changed their behaviour towards subsequently wearing a mouth guard, eight responded that it definitely had, three that it had to some degree, and two stated that it had not at all.

**Fig. 3:**
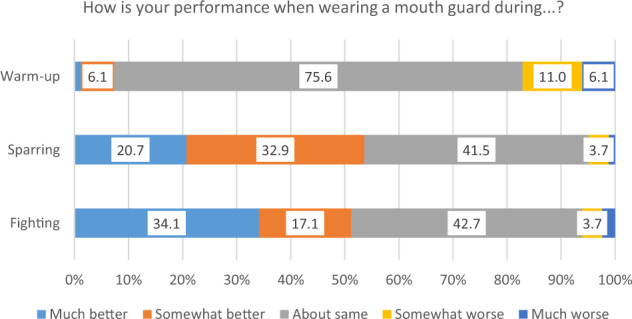
Stacked bar chart to show participant responses to the question ‘How is your performance when wearing a mouth guard during warm-up, sparring and fighting?’.

**Fig. 4:**
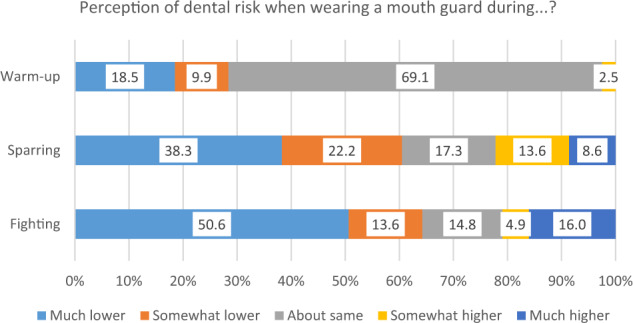
Stacked bar chart to show participant responses regarding their perception of dental risk when wearing a mouth guard during warm-up, sparring and fighting.

When asked about any impact of mouth guard use on their performance, most respondents (75.6%) considered there to be no impact during warm-up (Fig. 3). However, 53.6% and 51.2% of respondents considered their performance to be much better or somewhat better when wearing a mouth guard during sparring or fighting, respectively. Only a minority considered that wearing a mouth guard negatively impacted their performance during sparring (4.9%) or fighting (6.1%). The majority of respondents (69.1%) considered a mouth guard to not alter risk during warm-up, and 28.4% considered that dental risk would be reduced. Furthermore, most respondents agreed that a mouth guard lowers risk during sparring (60.5%) and fighting (64.2%) (Fig. 4). However, there was a sizeable percentage whose responses indicated that they considered wearing a mouth guard increases dental risk during sparring (22.2%) or fighting (20.9%). No statistically significant differences could be identified between these participants and those who considered that mouth guard use does not increase dental risk during sparring or fighting, either in respect of age, gender, skill level, or type of mouth guard worn (all *p* > 0.05).

Insufficient numbers of free text comments were recorded to permit formal qualitative analysis. However, a representative sample of free text comments is presented in Table 2. The majority of comments related to the type of mouth guard being used, with respondents indicating that they valued the benefits of a custom-made mouth guard compared to cheaper mouth-formed (‘boil and bite’) mouth guards in terms of comfort and fit.

**Table 2:** Representative free text participant comments.

Gumshields must be [made] to a certain quality as some boil and bite gumshields are shocking. I think it should [be] mandatory to wear custom fit dentist [-made ones] for anyone doing headshots … but all children should wear them (boil and bite ones at least) when competing, even when no headshots, just for safety in case of an accidental head shot. *[Male, age 25–29, advanced skill level]*
I got mine from an online business that sends a mould to your door, and you mould it yourself. It fits well and doesn’t move during blunt trauma. The boil and bite ones have always left me with cut gums due to the movement of the material over the gum line. *[Male, age 18–24, advanced skill level]*
I have always wanted to get a [custom] fitted gum guard. However, I have a semi-permanent retainer and have not wanted the expense to have the retainer removed. *[Male, age 18–24, advanced skill level]*
I tend to lose things as small as mouth guards quite easily. I wouldn’t mind paying £60 for a properly fitted mouth guard that would last 2 years, but if I lost that one (quite likely within 2 years) I would just replace it with a cheap one. *[Female, age 25–29, beginner skill level]*
I have, and have always had, the terribly bad habit of not wearing a mouth guard during sparring, despite knowing full well what risks this presents. This [is] usually down to the discomfort felt by low cost bite and boil mouth guards - but I should definitely invest in a [custom] fit [mouth] guard! *[Male, age 25–29, advanced skill level]*
I have had a number of mouth guards over the years, for Muay Thai as well as other sports such as American football and after getting a professionally fitted mouth guard from a dentist, I will never purchase a boil to fit mouth guard again. They are 100 times better. *[Male, age 18–24, advanced skill level]*
My mouth guard was made by a company not necessarily a dentist … was an easy way through the internet where you do the mould yourself and send it off. Best I’ve ever had. If your mouth guard is fitted on top and doesn’t fall down and you can speak clearly, I’ve noticed a massive difference. *[Female, age 25–29, professional skill level]*
I’ve never fully looked into whether there are any downsides to mouth guards, but from playing contact sports at school, going into boxing and then Muay Thai, it’s always been seen as standard practice. Whenever possible (i.e. when I have been able to afford to), I have gone for a mould mouth guard as I find it far more comfortable to wear and easier to communicate and breathe when wearing. *[Male, age 30–34, advanced skill level]*

## Discussion

Muay Thai is becoming an increasingly popular contact sport, and is seen by many participants as a good way to keep fit as well as building strength and confidence. Given that targeting the head and facial region with kicks and blows is an accepted part of the sport, then the risk for dental trauma must always remain high. We considered it important, therefore, to investigate the use of mouth guards in Muay Thai, as well as participants’ perceptions regarding their use.

The rate of dental injuries reported by participants in this study was 14% (13 respondents), with the most frequently reported injury being chipped or broken teeth. This is lower than that reported in previous studies; for example, in a study in Thailand, 24% of Muay Thai participants had experienced dental/jaw injuries,^12^ and in a study in Iran, 44% of combat sports participants had experienced dental injuries.^13^ It is possible that the use of mouth guards in the present study had helped to prevent dental trauma, resulting in a lower rate compared to previous studies, though we consider that even a lower rate of dental trauma is still too high, particularly if it might have been preventable.

Overall, in the present study, only 3% of the study participants reported never having worn a mouth guard. Furthermore, over 70% were extremely comfortable or very comfortable wearing a mouth guard during both sparring and fighting. Over 90% considered that protection, breathing, good fit and comfort were all extremely or very important when it came to their choice of mouth guard. Of the 13 individuals who had experienced dental injuries, eleven reported that this had either definitely changed or somewhat changed their attitude to wearing a mouth guard, and similar findings have been reported previously.^15^ Potentially, these individuals could be influential in increasing the uptake of mouth guard usage among those Muay Thai participants who do not yet perceive the benefits of wearing one.

It is notable that a greater proportion of participants (88%) wore a mouth guard during sparring than when fighting (61%). It is possible that participants considered issues such as the fit (or risk of movement) of the mouth guard and/or any impact on breathing to be potentially detrimental to their performance during a fight. Potentially linked to this finding is the observation that some participants considered wearing a mouth guard to increase dental risk during sparring (22%) and fighting (21%). This may again be related to experiences of discomfort or movement of (poor fitting) mouth guards during the high intensity situation of a fight. Further research is necessary to investigate this further, but we consider it to be an important finding that almost 40% of participants reported not wearing mouth guards during actual fights. This is a matter for concern, and we support previous calls that further education is needed for contact sports participants regarding the risks of dental trauma and the importance of wearing a mouth guard to protect the teeth.^16,17^

It is of interest that there was a statistically significant difference in the proportion of participants aged 18–29 years wearing mouth guards during a fight (73%) compared to those aged 30 years or older (50%). It may be that younger participants are more aware of the risks of dental trauma, or may have become used to wearing mouth guards from an early age as they participated in sporting activities at school. Alternatively, older fighters may have the perception that they are in control of risk, or may have become complacent as they acquired more experience in the sport.^10^ However, it has been reported that the risk for injury during Muay Thai is greater in more experienced participants,^9^ and clearly further research is needed to identify reasons why a greater proportion of older participants did not routinely wear a mouth guard. It may be that these participants are the group that would benefit most from an educational intervention, and perhaps an aspect which could be emphasised from the present study could be that only a minority of respondents considered that wearing a mouth guard negatively affected their performance, whether during sparring (4.9%) or fighting (6.1%).

We identified that the most frequently used type of mouth guards were mouth-formed (‘boil and bite’), being used by about 60% of participants, with around 32% wearing custom-made mouth guards from a dentist. A study of mouth guard use in grappling sports in the UK identified a similar pattern of mouth guard use, with twice as many participants wearing mouth-formed compared to custom-made mouth guards.^18^ Cost and convenience are likely to influence the decision on which type of mouth guard that individuals choose to use, and indeed, the great majority of those wearing mouth-formed mouth guards paid less than £20, compared to the custom-made mouth guards from a dentist which tended to cost around 2–3 times this amount. However, the benefits of a custom-made mouth guard from a dentist were definitely recognised by those participants who had followed this route, as indicated by the free text comments (Table 2). Although around half of the respondents in our study had spoken to their dentist about Muay Thai, the finding that only approximately one third were wearing a dentist-made mouth guard suggests it is worth encouraging dentists to be more pro-active in this regard when discussing sports or hobbies with patients, a suggestion that has also been made previously.^19^

The great majority of respondents (84%) agreed that mouth guards should be mandatory for children who participate in Muay Thai while at the same time, 62% considered that participants themselves should take the primary responsibility for mouth guard use. We consider that, at an organisational level, it is important that the use of mouth guards by children becomes mandatory, as has already been adopted on a local basis at individual gyms.

Limitations of our study include the higher than expected proportion of experienced participants, with almost 80% of respondents describing themselves as being at an advanced level, or coaches/professional fighters. It is possible that beginners chose not to respond to the survey, potentially feeling that they may not have sufficient experience to respond. Future studies could undertake purposive sampling to recruit a broader range of participants. Although our study was live for 6 weeks and collected 92 responses, this was slightly below the target. The number of responses was sufficient for descriptive analyses, but further detailed comparisons between sub-groups would require a greater sample size. A more qualitative approach in a future study may also help to identify issues around the reasons for not wearing mouth guards by certain individuals, so that appropriate educational interventions can be developed and piloted. Bias could also have been introduced in the present study by the use of an online questionnaire that primarily targeted participants at a specific gym. A broader approach ensuring better representativeness would be required in future studies.

## Conclusions

Whereas mouth guards were well tolerated and participants perceived the benefits of using a mouth guard for protection against injury, only 60.9% reported wearing them during fights. Furthermore, older participants were less likely to wear a mouth guard during fights when compared to younger participants. Whereas a relatively low proportion had experienced dental injury in Muay Thai (14%), it is still clear that participation in this contact sport presents a risk for dental trauma. In order to provide targeted advice for Muay Thai participants, we consider that dentists should be pro-active in recommending mouth guard use for contact sports participants, particularly custom-made mouth guards that are fabricated following impressions and are fitted in the dental clinic. Furthermore, it may be that those participants who have suffered dental injury in the past could be influential in increasing awareness of the risks of trauma, particularly in groups that do not routinely wear a mouth guard.
